# A Novel LncRNA MSTRG.310246.1 Promotes Differentiation and Thermogenesis in Goat Brown Adipocytes

**DOI:** 10.3390/genes14040833

**Published:** 2023-03-30

**Authors:** Jing Tang, Xin Liu, Duo Su, Tingting Jiang, Siyuan Zhan, Tao Zhong, Jiazhong Guo, Jiaxue Cao, Li Li, Hongping Zhang, Linjie Wang

**Affiliations:** 1 Key Laboratory of Livestock and Poultry Multi-Omics, Ministry of Agriculture and Rural Affairs, College of Animal and Technology, Sichuan Agricultural University, Chengdu 611130, China; 2 Farm Animal Genetic Resources Exploration and Innovation Key Laboratory of Sichuan Province, Sichuan Agricultural University, Chengdu 611130, China

**Keywords:** lncRNA, thermogenesis, goat, brown adipocyte, brown adipose tissue

## Abstract

Brown adipose tissue (BAT) plays a critical role in maintaining the body temperature in newborn lamb due to its unique non-shivering thermogenesis. Previous studies have found that BAT thermogenesis is regulated by several long non-coding RNAs (lncRNAs). Here, we identified a novel lncRNA, MSTRG.310246.1, which was enriched in BAT. MSTRG.310246.1 was localized in both the nuclear and cytoplasmic compartments. In addition, MSTRG.310246.1 expression was upregulated during brown adipocyte differentiation. Overexpression of MSTRG.310246.1 increased the differentiation and thermogenesis of goat brown adipocytes. On the contrary, the knockdown of MSTRG.310246.1 inhibited the differentiation and thermogenesis of goat brown adipocytes. However, MSTRG.310246.1 had no effect on goat white adipocyte differentiation and thermogenesis. Our results show that MSTRG.310246.1 is a BAT-enriched LncRNA that improves the differentiation and thermogenesis of goat brown adipocytes.

## 1. Introduction

Over the past decade, it has been widely reported that adaptive thermogenesis in adipose tissue could consume huge energy to treat obesity and other metabolic diseases. Adipose tissue is an endocrine organ with high metabolic activity. There are many classification methods for adipose tissue. According to the function of adipose tissue, they are divided into white adipose tissue (WAT), beige adipose tissue (BEG), and brown adipose tissue, while BAT and BEG are thermogenesis-specific types of adipose tissue [1,2]. BAT is highly recruited in newborn mammals for thermogenesis and plays a critical role in maintaining the body temperature in newborn mammals [3]. To maintain body temperature, the interscapular brown adipose tissue (iBAT) is preserved in adult mice [4]. However, the content of brown adipose tissue in goats reaches its maximum at birth, and then gradually disappears with age [5]. Thermogenesis of BAT is regulated by many factors, of which, sympathetic nerve is an important transfer station. After receiving the signal from the sympathetic nerve, brown adipose tissues generate heat through non-shivering thermogenesis (NST) and thus respond to cold stimulation [6]. Uncoupling protein 1 (UCP1) is a transmembrane protein, which is located in the inner mitochondrial membrane. It allows protons to flow freely through the inner membrane of mitochondria, which causes the electron transport chain to be uncoupled from oxidative phosphorylation. Finally, the energy in the proton gradient is dissipated in the form of heat, and such a process is essential for brown and beige adipocyte thermogenesis [7,8,9]. In our previous study, we identified that the UCP1 in goats reaches its highest expression at birth and then disappears at day 7 after birth [5]. This is why we say that the thermogenesis of BAT is crucial in newborn lambs. Furthermore, there are several molecules that can regulate the physiology of BAT thermogenesis or be used as biomarkers of BAT development. PPARG coactivator 1 alpha (PGC1α) is a key transcriptional coactivator that regulates BAT thermogenesis, which promotes the expression of thermogenesis-related genes [10]. Additionally, peroxisome proliferator-activated receptor alpha (PPARA), carnitine palmitoyltransferase 1A (CPT1A), ELOVL fatty acid elongase 6 (ELOVL6), and cell death-inducing DFFA-like effector A (CIDEA) usually serve as marker genes for brown adipocyte thermogenesis [11,12].

In mammals, adipose tissue possesses various functions, including insulin sensitivity, storing energy, glycolipid metabolism, and others. The high mortality of newborn piglets is due to their inability to maintain body temperature. Overexpression of PRDM16 in porcine adipocytes proved that porcine adipocytes have the potential of browning [13]. However, as reported, pig lacks the complete UCP1 gene [14]. Co-overexpressing the mice UCP1 and pig PGC1α in porcine adipocytes significantly increases the number of mitochondria in adipocytes and increases their uncoupled respiration rate, indicating that the browning of adipocytes plays an important role in improving the thermogenesis of piglets [15]. Previous studies demonstrated that lipid droplet (LD) in BAT is necessary for the cold-induced non-shivering thermogenesis, but researchers have found that the LD lipolysis in brown adipocytes is not necessary under cold stimulation by knocking out the key genes of lipolysis in adipocytes [16]. These results suggest that, although BAT is adipose tissue, it may regulate the metabolism of the body by regulating other metabolites. Further research found that in addition to thermogenesis, BAT also regulates the systemic glucose homeostasis. For example, the transport of glucose to white adipocytes under the stimulation of insulin is destroyed by the specific knockout of Rab10, a protein solely from brown adipocytes, which participates in the uptake of glucose to white adipocytes under the stimulation of insulin. The results are significantly related to the systemic glucose intolerance and insulin resistance, indicating that brown adipocytes participate in the systemic metabolic homeostasis of mice under the stimulation of insulin [17]. Previous studies on humans have also shown that activation of β3-adrenergic receptors (β3-ARs) promotes the browning of human subcutaneous WAT, improving the insulin sensitivity and glucose homeostasis [18]. Moreover, BAT also plays an important role as an endocrine organ. For instance, C-X-C motif chemokine ligand-14 (CXCL14), a BAT-secreted brown adipokine, promotes the browning of WAT and improves glucose homeostasis [19]. Adiponectin is a hormone protein secreted by adipocytes. Studies found that the expression of adiponectin was significantly related to the lactation traits and meat quality traits of cattle [20,21].

LncRNAs are RNAs that lack the ability to code proteins at a length of more than 200 nt [22]. LncRNAs are divided into sense, antisense, intergenic, and intronic lncRNAs [22,23]. Additionally, lncRNAs widely exist in various tissues of eukaryotes and have a certain spatial and temporal expression pattern. However, the conservation of lncRNAs among species is low; therefore, they often do not form large homologous families [23]. Furthermore, lncRNAs are involved in multiple physiological regulations, such as epigenetic regulation, dose compensation effect, and cell differentiation regulation [24,25,26,27]. Moreover, several studies found that lncRNAs regulate BAT thermogenesis through different regulatory mechanisms [28,29]. For example, PGC1β-OT1 is a lncRNA at a length of 1759 nt and inhibits adipogenic differentiation through antagonizing miR-148a-3p and enhancing the KDM6B expression [30]. In common with PGC1β-OT1 is TCONS_00023297, a novel lncRNA that promotes osteogenic differentiation through binding to miR-608 to further regulate the differentiation of bone marrow mesenchymal stem cells (hBMSC) [31]. Blnc1 formats the Blnc1/hnRNPU/EBF2 ribonucleoprotein complex to promote the expression of the thermogenic genes in brown adipocytes [32]. AK079912 regulates the browning of white adipocytes by affecting the mitochondrial copy number and the expression levels of the electron transfer chain-related proteins [33]. FOXC2-AS1 is an antisense lncRNA that induces the browning of white adipocytes through the autophagy signaling pathway [34].

Although several lncRNAs have been characterized to regulate BAT thermogenesis, the functions of many other BAT-enriched lncRNAs remain unclear. In a previous study, we identified a total of 249 BAT-enriched lncRNAs in goats by RNA-seq [5]. However, the functions of these BAT-enriched lncRNAs remained unknown. In this study, we investigate whether the novel lncRNA-MSTRG.310246.1 contributes to the development and thermogenesis of goat brown adipocytes. We found that MSTRG.310246.1 promoted the differentiation and thermogenesis of goat brown adipocytes, but not in white adipocytes.

## 2. Materials and Methods

### 2.1. Animals

All animal procedures were approved by the Institutional Animal Care and Use Committee at the College of Animal Science and Technology, Sichuan Agricultural University, Sichuan, China. Chuanzhong black goats at 1 day (*n* = 6) and 30 days after birth (*n* = 6) were raised at the breeding center of Sichuan Agricultural University, Ya’an, China. Under full anesthesia, goats were sacrificed by arterial bleeding. Then, perirenal adipose tissues of goats were collected for brown preadipocytes and white preadipocytes isolation. To detect tissue expression patterns, the different tissues of goats at 30 days after birth (*n* = 6) were collected and stored at −80 °C.

### 2.2. Total RNA Extraction and qPCR

Cell Total RNA isolation kit (FOERGENE, Chengdu, China) was used to extract total RNA, and then reverse-transcribed into cDNA by Reverse transcription kit (ABclonal, Wuhan, China). Genious 2x SYBR Green Fast qPCR mix (ABclonal, Wuhan, China) was used to perform qPCR and detected by Bio-Rad CFX96 qPCR instrument (Bio-Rad, Hercules, CA, USA). In a previous study, we found that *PFDN5* and *TBP* genes were the most stable reference genes for BAT to WAT transformation through RNA-seq [35]. Thus, *TBP* and *PFDN5* genes were applied as reference genes. Primers used in this study were shown in Table S1. The relative expression levels of the target genes were normalized relative to the expression of *PFDN5* and *TBP* using the 2^−ΔΔCT^ method. Six biological replicates and three technical replicates in a biological sample were used for qPCR.

### 2.3. Bioinformatics Analysis

The sequence alignment was processed using ENSEMBL (https://asia.ensembl.org/index.html), accessed on 6 February 2022. Coding Potential Calculator 2 (http://cpc2.gao-lab.org/) was used to evaluate the coding potential of MSTRG.310246.1, accessed on 16 February 2022 [36].

### 2.4. Cytoplasmic and Nuclear RNA Extraction

Brown adipocytes were collected at the proliferation of 80% fusion and sixth day of differentiation, respectively. Then, Cytoplasmic and Nuclear RNA purification kits (Norgen, Thorold, ON, Canada) were used to extract the RNA of the cytoplasm and nucleus. Briefly, cells were lysed by lysis buffer for 5 min. Lysate was spun for 10 min at maximum speed in a benchtop centrifuge, cytoplasmic RNA was in the supernatant and nuclear RNA was in the pellet. RNA was then reverse transcribed to cDNA and conducted qPCR as previously described. *GAPDH* was applied as the reference gene of the cytoplasm and *U6* was applied as the reference gene of the nucleus. Primers used in this are shown in Table S1.

### 2.5. Primary Goat Brown Adipocytes Culture

Primary brown adipocytes were obtained from perirenal adipose tissues at 1 day after birth of goats. Briefly, the adipose tissues were digested using 2 mg/mL collagenase (Sigma, St. Louis, MA, USA) at 37 °C for 25 min. Then, the cell suspension was filtered through 70 μm filters. Then, they were centrifuged at 1500 rpm/min for 5 min to isolate preadipocytes. The preadipocytes were cultured in a growth medium containing DMEM/F-12, 10% FBS, and 2% Penicillin/Streptomycin at 37 °C with 5% CO_2_ for 5 days.

For adipogenic differentiation of primary brown adipocytes, cells were treated with a medium containing DMEM/F-12, 5 μg/mL insulin (Solarbio, Beijing, China), 1 μM dexamethasone (DEXA) (Sigma, St. Louis, MA, USA), 0.5 mM 3-isobutyl-methylxanthine (IBMX) (Sigma, St. Louis, MA, USA), 1 μM rosiglitazone (Sigma, St. Louis, MA, USA), and 1 nM T3 (Selleck, Houston, TX, USA) for 2 days. After induction, cells were cultured with a maintenance medium containing DMEM/F-12, 5 μg/mL insulin, and 1 nM T3 for 6 days.

### 2.6. Primary Goat White Adipocytes Culture

Primary white adipocytes were obtained from perirenal adipose tissues at 30 days after the birth of goats. The adipose tissues were digested using 2 mg/mL collagenase (Sigma, St. Louis, MA, USA) at 37 °C for 70 min. Then, the same protocol as the separation of brown preadipocytes was followed. Cells were cultured with a growth medium containing DMEM/F-12, 10% FBS, and 2% Penicillin/Streptomycin at 37 °C with 5% CO_2_ until 100% confluence.

For adipogenic differentiation of primary white adipocytes, cells were treated with a medium containing DMEM/F-12, 5 μg/mL insulin (Solarbio, Beijing, China), 1 μM dexamethasone (DEXA), 0.5 mM 3-isobutyl-methylxanthine (IBMX) (Sigma, St. Louis, MA, USA), 125 μM indomethacin (Selleck, Houston, TX, USA), and 1 nM T3 (Selleck, Houston, TX, USA) for 2 days. After induction, cells were cultured with a maintenance medium containing DMEM/F-12, 5 μg/mL insulin, and 1 nM T3 for 6 days.

### 2.7. Plasmid Construction and Transfection

To construct the MSTRG.310246.1 overexpression of plasmids, the MSTRG.310246.1 was amplified and then ligated to pCDNA3.1 vector. The pCDNA3.1-MSTRG.310246.1 were transfected into brown and white adipocytes using lipofectamine 3000 (Invitrogen, Carlsbad, CA, USA) at the third day of differentiation. For knockdown of MSTRG.310246.1, we designed the small-interfering RNA (siRNA) to specifically silence MSTRG.310246.1. MSTRG.310246.1 siRNA oligonucleotides, Si-1: CTGCACTTCTGAACTTGAA; Si-2: CAGACAGTGTAATACTACT.

### 2.8. Oil Red O Staining

Adipocytes were fixed with 4% paraformaldehyde for 30 min. After fixed adipocytes, adipocytes were infiltrated with 60% isopropanol for 2–3 min. Then, adipocytes were stained with the oil red O solution at 37 °C for 30 min. Finally, isopropanol was used to dissolve oil red O and the OD values were measured at 510 nm.

### 2.9. Statistical Analysis

Data are presented as mean ± SEM. The one-way ANOVA was performed by Graphpad Prism 7 (Graphpad, San Diego, CA, USA), and Duncan’s new multiple range tests were used to analyze statistical significance. * *p* < 0.05, ** *p* < 0.01 were considered to be statistically significant.

## 3. Results

### 3.1. Identification and Characterization of MSTRG.310246.1 in Goat Brown Adipocytes

**Figure 1:**
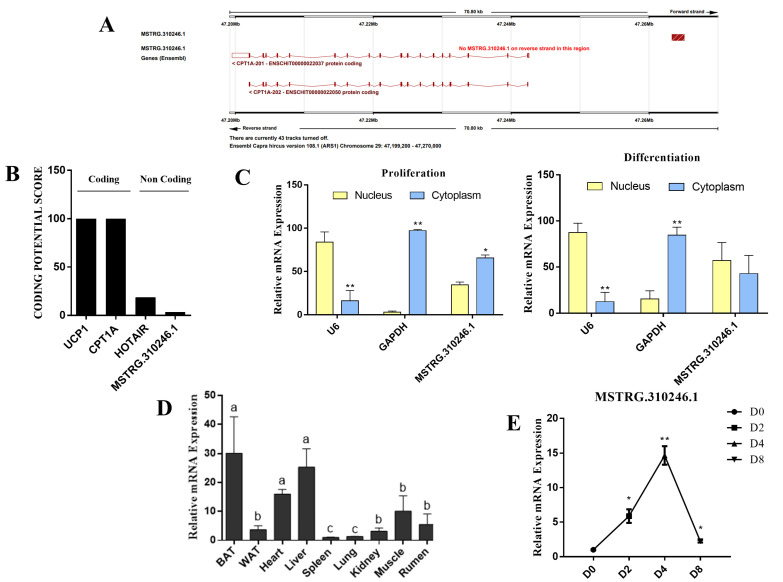
Identification and characterization of MSTRG.310246.1 in goats. (**A**) Position of MSTRG.310246.1 on the goat genome. (**B**) Coding ability prediction of MSTRG.310246.1. (**C**) Nuclear and cytoplasmic localization results showed that MSTRG.310246.1 was localized in both the nuclear and cytoplasmic compartment in the proliferation and differentiation stages of brown adipocytes. (**D**) The expression of MSTRG.310246.1 in BAT, WAT, heart, liver, spleen, lung, kidney, muscle, and rumen of goats. Error bars represent SEM of six biological replicates, *n* = 6. The different letters (a,b,c) in the histogram revealed that MSTRG.310246.1 expressed significantly different among each other (*p* < 0.05). (**E**) The expression level of MSTRG.310246.1 in different differentiation stages of goat brown adipocytes. Error bars represent SEM of six biological replicates, *n* = 6, * *p* < 0.05, ** *p* < 0.01 relative to D0 level.

MSTRG.310246.1 was located on goat chromosome 29 at a length of 1817 nt and had one exon structure (Figure 1A and Table S2). Then, we used Coding Potential Calculator 2 to predict the coding ability of MSTRG.310246.1. As shown in Figure 1B, UCP1 and CPT1A genes have coding ability as negative control and HOTAIR is a long noncoding RNA as a positive control. The coding score of MSTRG.310246.1 was extremely lower than UCP1 and CPT1A genes, indicating that MSTRG.310246.1 lacks the ability to code protein (Figure 1B). qPCR analyses of fractionated nuclear and cytoplasmic RNA indicated that MSTRG.310246.1 was mainly localized in a cytoplasmic compartment in the proliferation stage and in both the nuclear and cytoplasmic compartment in the differentiation stages of brown adipocytes (Figure 1C). MSTRG.310246.1 expression was significantly higher (*p* < 0.01) in BAT, liver, and heart than that in other tissues (Figure 1D). There was no significant expression difference among BAT, liver, and heart. In addition, MSTRG.310246.1 expression was upregulated in brown adipocytes during adipogenesis, and reached the peak at day 4 of differentiation (*p* < 0.01), and then decreased at day 8 (Figure 1E).

### 3.2. Overexpression of MSTRG.310246.1 Induced the Differentiation and Thermogenesis of Goat Brown Adipocytes

**Figure 2:**
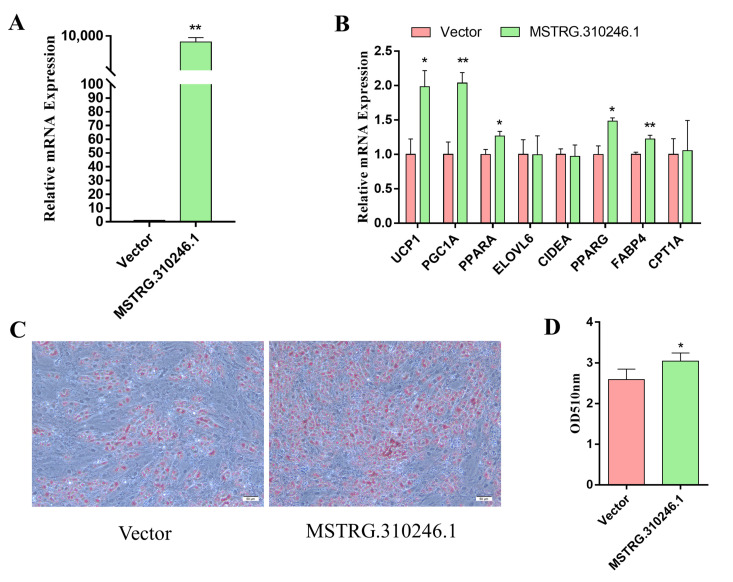
Overexpression of MSTRG.310246.1 induced the differentiation and thermogenesis of brown adipocytes. (**A**) Overexpression of MSTRG.310246.1 in goat brown adipocytes. (**B**) Gene expression in goat brown adipocytes transduced with MSTRG.310246. (**C**,**D**) Oil-red-O staining of brown adipocytes transduced with MSTRG.310246.1. Bar: 50 μm. The absorbance at 510 nm was detected. Error bars represent SEM, *n =* 6, * p < 0.05, ** p < 0.01.

To explore the function of MSTRG.310246.1 in adipogenesis and thermogenesis, we transferred pCDNA3.1-MSTRG.310246.1 plasmid into brown adipocytes. Overexpression significantly increased the expression level of MSTRG.310246.1 (*p* < 0.01) (Figure 2A). Furthermore, overexpression of MSTRG.310246.1 upregulated the adipogenic gene expression of *PPARG* (*p* < 0.05) and *FABP4* (*p* < 0.01) (Figure 2B). To further detect the differentiation of brown adipocytes, oil red O was used to stain the cells and the OD value was measured at 510 nm. These results showed that the triglyceride (TG) content in brown adipocytes was increased in cells overexpressing MSTRG.310246.1 (*p* < 0.05) (Figure 2C,D), suggesting that MSTRG.310246.1 plays important roles in the differentiation of brown adipocytes. Furthermore, overexpression of MSTRG.310246.1 also increased the BAT thermogenic gene expression of *UCP1* (*p* < 0.05), *PGC1A* (*p* < 0.01), and *PPARA* (*p* < 0.05) (Figure 2B). These results indicated that MSTRG.310246.1 enhanced the goat brown adipocytes differentiation and promoted the expression of BAT-thermogenesis-related genes.

### 3.3. MSTRG.310246.1 Knockdown Inhibited Brown Adipocyte Differentiation and Thermogenesis

**Figure 3:**
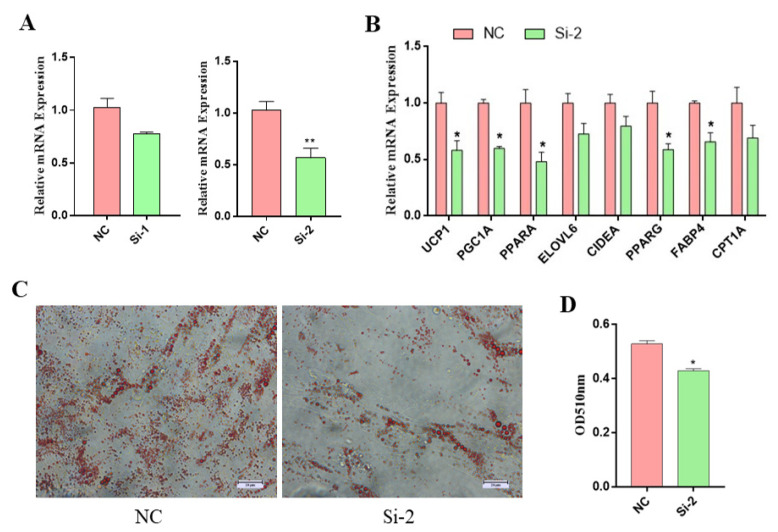
MSTRG.310246.1 knockdown inhibited the differentiation and thermogenesis of brown adipocytes. (**A**) Knockdown of MSTRG.310246.1 in brown adipocyte. (**B**) Gene expression in brown adipocytes transduced with NC or siMSTRG.310246. (**C**,**D**) Oil-red-O staining of brown adipocytes transduced with NC or siMSTRG.310246. Bar: 20 μm. The absorbance at 510 nm was detected. Error bars represent SEM, *n =* 6, * *p* < 0.05, ** *p* < 0.01.

Next, we used two small-interfering RNAs (siRNAs) to silence MSTRG.310246.1 expression in brown adipocytes. We found that siRNA-1 (Si-1) has no inhibition on the expression of MSTRG.310246.1. The silencing of MSTRG.310246.1 with siRNA-2 (Si-2) markedly reduced the expression of MSTRG.310246.1 in brown adipocytes (*p* < 0.05) (Figure 3A). MSTRG.310246.1 knockdown led to a decreasing expression of adipogenic gene expression of *PPARG* (*p* < 0.05) and *FABP4* (*p* < 0.05) (Figure 3B). Through oil-red-O staining analysis, we found that the lipid accumulation was significantly decreased by MSTRG.310246.1 knockdown (Figure 3C,D). Moreover, knockdown of MSTRG.310246.1 decreased the expression of BAT marker genes, including *UCP1* (*p* < 0.05), *PGC1A* (*p* < 0.05), and *PPARA* (*p* < 0.05) (Figure 3B). Consistently, MSTRG.310246.1 knockdown inhibited the differentiation and suppressed the expression of BAT-thermogenesis-related genes of goat brown adipocytes.

### 3.4. MSTRG.310246.1 Had No Effect on Goat White Adipocytes Differentiation and Thermogenesis

**Figure 4:**
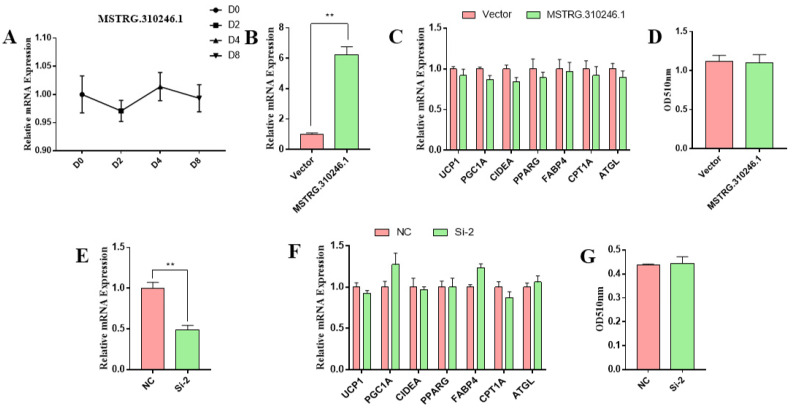
MSTRG.310246.1 had no effect on differentiation and thermogenesis in white adipocytes. (**A**) The expression pattern of MSTRG.310246.1 during the differentiation of white adipocytes. (**B**) Overexpression of MSTRG.310246.1 in white adipocytes. (**C**) Gene expression in white adipocytes transduced with MSTRG.310246. (**D**) The absorbance at 510 nm of oil-red-O staining of brown adipocytes transduced with vector or MSTRG.310246. (**E**) Knockdown of MSTRG.310246.1 in white adipocyte. (**F**) Gene expression in white adipocytes transduced with inhibitor NC or MSTRG.310246 inhibitor. (**G**)The absorbance at 510 nm of oil-red-O staining of brown adipocytes transduced with inhibitor NC or MSTRG.310246 inhibitor. Error bars represent SEM, *n =* 6, ** *p* < 0.01.

We firstly determined the expression of MSTRG.310246.1 in white adipocytes, and the result showed that there was no significant difference in the expression of MSTRG.310246.1 during white adipocytes differentiation (Figure 4A). To further determine whether MSTRG.310246.1 regulates the expression of thermogenic and adipogenic genes in goat white adipocytes, pCDNA3.1-MSTRG.310246.1 was transfected into white adipocytes, and MSTRG.310246.1 expression was significantly elevated (*p* < 0.01) (Figure 4B). However, adipogenic markers did not show any significant changes compared with the control group, including PPARG and FABP4 (Figure 4B). We then detected the expression of BAT-thermogenesis-related genes, and there is no remarkable difference between control and treatment group (Figure 4C). In addition, the content of TG characterized by oil-red-O staining also showed no significant difference by the overexpression of MSTRG.310246 (Figure 4D).

Next, we performed loss-of-function studies of MSTRG.310246.1 to determine the significance of MSTRG.310246.1 in white adipocyte differentiation. Interfering MSTRG.310246.1 significantly decreased MSTRG.310246.1 expression levels of white adipocytes (*p* < 0.01) (Figure 4E). Nevertheless, the expression levels of thermogenic and adipogenic genes were not changed in MSTRG.310246.1-knockdown white adipocytes (Figure 4F). In addition, silence of MSTRG.310246.1 did not result in significant changes to lipid deposition (Figure 4G). These results suggested that MSTRG.310246.1 had no effect on white adipocyte differentiation and thermogenesis.

## 4. Discussion

As a thermogenic adipose tissue, BAT plays an important role in the treatment of obesity, diabetes, and other related diseases [37]. The thermogenesis of BAT is essential for mammals to adapt to changes in temperature at newborn stages. In sheep and goats, the content of BAT gradually disappears with age. This is consistent with the expression of UCP1 reaching its highest at birth and then disappearing gradually, which is one of the marker genes of BAT [38]. Several studies have explored the developmental regulation of BAT in goats. The content of BAT is relatively small and mainly distributed in the perirenal, clavicular, and pericardiac regions. Previous studies on sheep and goats have found that the perirenal adipose tissue is BAT at birth and then changes into WAT with age [5,39]. Studies found that the sternal fat of female sheep presents the characteristics of brown adipose tissue [4]. For adult goats and sheep, white adipose tissue with browning ability can be induced into beige adipose tissue under cold or metabolic imbalance, thus providing heat for the body or regulating metabolic balance [40]. Cold exposure significantly increased the expression of BAT thermogenesis genes in newborn goats and increased the level of metabolites involved in the metabolism of glycerol phospholipids and glycerol phospholipids [41]. We previously found that 157 metabolites (76 down-regulated and 81 up-regulated) changed significantly during the transformation from BAT to WAT, among them, L-carnitine was considerably enriched in BAT [42]. L-carnitine is an intermediate of fatty-acid degradation and is necessary for goat brown adipocyte differentiation and thermogenesis. Moreover, maternal L-carnitine supplementation increased the rectal temperature of newborn goats and promoted BAT thermogenesis. Further research found that L-carnitine promoted TG and glycogen deposition in brown adipocytes through AMPKα [43].

As a multifunctional regulator, lncRNA has been confirmed to play an important role in the formation and differentiation of brown adipose tissue [44]. Although the regulation mechanism of lncRNA is varied, the final result was mainly to change the expression of adipose-related genes and thus to regulate the formation and differentiation of adipose tissue. Among them, lnc-BATE1 is the key to establishing and maintaining the BAT morphology and its thermogenic capacity. It mediates the transactivation of brown adipose tissue and the inhibition of white adipose-related genes [45]. LncRNA-AK079912 is a brown adipocyte-enriched lncRNA, which promotes the differentiation and thermogenesis of adipocytes through PPARG [33]. Moreover, uc.417 is a lncRNA transcribed from an ultraconserved region in rodents and the expression of uc.417 is related with age. Studies found that uc.417 inhibited the phosphorylation of MAPK, which is crucial for the expression of UCP1 [46]. In the previous study, we conducted a differential analysis of noncoding RNA between BAT and WAT in goats, and identified a total of 27 BAT-enriched circRNAs, 249 BAT-enriched lncRNAs, and 167 BAT-enriched miRNAs [5,47,48]. Furthermore, we found that miR-433 inhibits MAPK8 expression by targeting 3’UTR of MAPK8, indicating that miR-433 negatively regulates brown adipocytes’ thermogenesis [48]. However, the functions of many BAT-enriched lncRNAs remain unclear. 

Our previous study found that MSTRG.310246.1 was enriched in BAT compared with WAT by RNA-seq [5]. In this study, we found that MSTRG.310246.1 is a BAT-enriched lncRNA, which promoted differentiation and thermogenesis in brown adipocytes but not in white adipocytes of goats. However, the regulation mechanism of MSTRG.310246.1 on brown adipose tissue thermogenic function still needs to be investigated. As MSTRG.310246.1 is a BAT-enriched lncRNA, we performed the experiments in brown adipocytes and white adipocytes, respectively. In brown adipocytes, we found that MSTRG.310246.1 induced the expression of thermogenic genes and TG deposition. Furthermore, when we conducted the loss-of-function experiment of MSTRG.310246.1 in brown adipocytes, we got the opposite result of overexpression, indicating that MSTRG.310246.1 promoted the differentiation and function of brown adipocytes. In white adipocytes, we showed that MSTRG.310246.1 did not regulate the differentiation or browning of white adipocytes. Overexpression or knockdown of MSTRG.310246.1 in white adipocytes did not change the expression of thermogenesis-related genes and TG deposition. These results suggest that MSTRG.310246.1 plays an important role in the differentiation and function of brown adipocytes. However, the regulation mechanism of MSTRG.310246.1 on brown adipose tissue thermogenic function still needs to be investigated.

## 5. Conclusions

In this study, we characterized MSTRG.310246.1 as a new brown adipocyte regulatory factor, which had a limited role in goat white adipocytes, but played an important role in goat brown adipocytes. In support of this notion, overexpression and knockdown studies showed that MSTRG.310246.1 promoted the differentiation and thermogenesis of goat brown adipocytes, but had no effect on white adipocytes.

## Data Availability

No new data were created or analyzed in this study. Data sharing is not applicable to this article.

## Supplementary Materials

The following supporting information can be downloaded at: https://www.mdpi.com/article/10.3390/genes14040833/s1, Table S1: Primers used for real-time RT/PCR. Table S2: The full length of goat MSTRG.310246.1.
