# Supplementary material for: A Novel LncRNA MSTRG.310246.1 Promotes Differentiation and Thermogenesis in Goat Brown Adipocytes
Source: Genes (Basel). 2023 Mar 30;14(4):833. doi: 10.3390/genes14040833 (PMC10137646; doi:10.3390/genes14040833)
Supplement: Supplementary file 1 [file genes-14-00833-s001.zip › Table S1.pdf]

Table S1. Primers used for real-time RT/PCR.

| **Gene symbol** | **Sequence 5′-3′** | **Tm(℃)** | **Size(bp)** |
|---|---|---|---|
| UCP1 | TCCTGTCTTTGATCGCCTCT<br>GAACAGTCCATGTGCCAGTG | 61.3 | 135 |
| PGC1α | CCACAAATGATGACCCTC<br>GGTTTGGCTTGTAGATGTT | 60 | 103 |
| ELOVL6 | TGTCAGAGATGACAAGGGCT<br>CTTGGTTCGCACGGATTCTC | 62.5 | 121 |
| PPARγ | GTGTCACTCCTGAACGAAAT<br>GGAAATGCTGGAGAAGTCAA | 60 | 156 |
| PPARα | TGCAAGCTTGGACTTGAACG<br>ATGAAGCCATTCCCGTAGGC | 60 | 126 |
| FABP4 | ACTGGGATGGGAAATCAACC<br>CCTTGGCTTATGCTCTCTCG | 60 | 117 |
| CPT1α | CGTCGCCTTCCAGTTCACAG<br>CACAACCACGATGAGCCAACT | 53.7 | 181 |
| ATGL | TCCTGAGAACTTCGCACCTG<br>GCAGCCACACACAAGTGAAG | 60 | 142 |
| CIDEA | TCAAGAGTGGATAGGGGGCA<br>CCAGTCCCTGATAGTCGCTC | 60 | 147 |
| Full length of MSTRG.310246.1 | CGGGATCCCCACTCACTCTTGCCAGCTA<br>ATAAGAATGCGGCCGCTGTCATGCAGTGTGGCA | 63 | 1841 |
| MSTRG.310246.1 | GCTCCCTGCATGTTAAGCCT<br>GGCATCTGCTAATGTGTGCC | 62.5 | 142 |
| U6 | GGAACGATACAGAGAAGATTAGC<br>TGGAACACTTCTGGAATTTGCA | 60 | 68 |
| GAPDH | GCAAGTTCCACGGCACAG<br>GGTTCACGCCCATCACAA | 61.3 | 249 |
| PFDN5 | GCTTATTGACGTGGGAACT<br>TGCAGAGCTGGCTGGATT | 60 | 120 |
