# Supplementary material for: A Novel LncRNA MSTRG.310246.1 Promotes Differentiation and Thermogenesis in Goat Brown Adipocytes
Source: Genes (Basel). 2023 Mar 30;14(4):833. doi: 10.3390/genes14040833 (PMC10137646; doi:10.3390/genes14040833)
Supplement: Supplementary file 1 [file genes-14-00833-s001.zip › Table S2.pdf]

Table S2. The full length of goat MSTRG.310246.1

CCACTCACTCTTGCCAGCTACATTTCTCGCTGGCATCTCCCAGCAGGTTCTGATCCAAA

AGTGTGTGAGATTTTTGGGAACTCAGTTTCCATTTCCCAATGGAAGAATTTTGTAATCT

CTGGGTTCTGCACTTCTGAACTTGAACTTTGAACCCTGTAGGCAGTGAGTAAACAGTG

ACAGTTACACGTCAGGGTAGTGACAGTGTTTGCTTGGCCACCATCACTGGGGTTTGGT

AAAGGCAAAATGAAGTAATGCAGATGCAAGCAAAGAAGGAAGGCAGCTAATCCATGT

GTGGGTGAAAGAAGCTCGGAGTGAGAGCATTATCCTACTCCTGCACATGCAACAACAA

CAACAACAAAATGAGAGCCCCCGTCCTGGAAAAGACAGATGCGTGCAAGAATGTTTAT

GGAAGATTCACTTCAGGTGATAAAAATCCAGAAACAACCCAGATACCCAGCAGCAATA

GCATGGGTAAATAAATCGTGGTTTCGCCAGACAGTGTAATACTACTCAGAAAAAAGTA

AACTACGGACACATGCAACATGGGTAGATCTTACAGATGTTATGTTGAGCAAAAGGAA

GCCAAACAGACACACAAAAGTAAATATACCGCATGATTTCAATAACAGAAGTGAGGCT

TATTGGTGGCAGTGATGATAATCACTAGAAATACACAAAGAAGCCCCCAGAGTGGGCG

TGTGTGTGAAAGTGCATCGCTCTGTATGCTTAAGATTATACTCTTAACAATCTTTGCTCC

CTGCATGTTAAGCCTCAATTTTAAGAAATCAAAACTTAAAAAGAGATCCTTGATGTCGA

GATCTTGACAGGCCTGCCTGGACACTGGGACCTCAAAGACCAGAAAGGACTGTCCTG

CGGCACACATTAGCAGATGCCTGAATATGCAAATGAGAGCACTTACGGAAGACTTCAG

TGTCAAGATGCTAATTCTTCCGCTTCGGAGAAGGAGCTGGGGAGGTCAATAGCTTTTG

AGTGGACAGTTTTTAAACTTGGGGGAGGCTGGTTCACTGACCCTTTGAAAAGCTGATG

ACAGCTGCAAACCTTTTTTTTTTTTCCAGAAAAATGCACACACACCTAAAGAGTGGTA

AAGAATATCAGTCTTGGAGCTTCCTAAGCCCTTGTACCAACTCCTGACCCACATAGATT

AAGAAAAATGTATCCGCAATTTTGGAACAAGGTAAAAGGATATCTGAAATGTCCTTTCA

CTGGCTCACTGTCTAACAGGCCGCCCAGGGATTAGCTCTTTCTCTAGGGATTTGTGTCT

TGAATAAGCTCTAGATGCTACAAAGTTACATGTAACTGGTAGATTTTTGGCCAACAAAA

ATGCAAGGGTTAAAACAGATCACAAATAAGGACCCACCTACAGCACACGGAACTCTAC

TCAGTACTCTGTAATGGCCTATATGGGGAAAGAATCTAAAAAAGAGTGGATATATGTATA

TGTATAACTGATTCACTTTGCTGCTCACCTGAAATAACGCAACATTGTAAATCAGCTATA

TGCCAATAAAACTTTTTAAAAAAGAAAAAGAAATGCAAGGATTTCTATTGGTTGAAAA

AAAGGATTTTCCCCAAAGTTGTGGTTTTGTGGCCCCGTAGAATATTAACCAGTTTTTA

AATCTATTTAGGAAAATCAGATTTTTGTCTATCTTCCTGTGCAGGGAAACCTCCAGCTCT

TCCCCATTGTATATTTCTTCTTGTTTGTTTTCTTTACTGCTCACAGGAAATATTTCACTTT

CTTTTGGAAAGAATTTTCTGGTTTAAAACTTTATTTATTTATTTGCCACACTGCATGACA
